# Studies on the cholinesterases inhibiting compounds from the Cassiopea andromeda venom

**DOI:** 10.6026/97320630016702

**Published:** 2020-09-30

**Authors:** Amir Hossein Darabi, Iraj Nabipour, Gholamhossein Mohebbi, Amir Vazirizadeh, Hossein Vatanpour, Ammar Maryamabadi

**Affiliations:** 1 The Persian Gulf Tropical Medicine Research Center, The Persian Gulf Biomedical Sciences Research institute, Bushehr University of Medical Sciences, Bushehr, Iran; 2 The Persian Gulf Marine Biotechnology Research Center, The Persian Gulf Biomedical Sciences Research Institute, Bushehr University of Medical Sciences, Bushehr, Iran; 3 Department of Marine Biotechnology, The Persian Gulf Research and Studies Center, The Persian Gulf University, Bushehr, Iran; 4 Department of Pharmacology and Toxicology, Faculty of Pharmacy, Shaheed Beheshti Medical Sciences University, Tehran, Iran

**Keywords:** Cassiopea andromeda, Venom, Cholinesterases, in vitro, in silico, Alzheimer's disease

## Abstract

Cholinesterase inhibitors find application in the combat and care of several diseases, especially AD. Jellyfish venoms are the most promising sources of potent cholinesterase inhibitors. Therefore, it is of interest to study cholinesterases inhibiting compounds from the Cassiopea andromeda venom. We report bioactive compounds using the GC-MC method followed by molecular modeling and docking data analysis. The GC-MS analysis of the crude venom led to the identification of seven bioactive compounds (C1-C7), comprising the steroidal alkaloids, phenolic and carotenoid derivatives. The venom exhibited inhibitory activities against the cholinesterase enzymes. The compound C2, a Dioxolane steroid, displayed the strongest inhibition on both AChE and BChE activities for further consideration.

## Background

Alzheimer's disease (AD) is a neurodegenerative disease and the most cause of dementia [1]. AD is characterized by an insidious decrease or loss in memory, personality alteration, and decline of cognitive and non-cognitive functions, which thoroughly leads to disabling of the patient [2]. Its pathogenesis is related to the loss of cholinergic neurons, subsequently, a decline in acetylcholine (ACh). It seems to be produced by either reduced choline transferase or enhanced AChE activities [3].

AChE is a serine hydrolase belonging to the carboxylesterase family of enzymes, which ACh break down into choline and acetate [4]. The most significant attribute of AChE is a deep and confined gorge. The catalytic site of the enzyme is positioned at this gorge known as the catalytic triad, which is consisted of His447, Glu334, and Ser203. In this part, the neurotransmitter is hydrolyzed. A peripheral site composed of several aromatic site chains that extend beyond Tyr337 at the catalytic/peripheral site interface to the entrance of gorge contributes to the AChE catalytic efficiency. The peripheral site comprises Tyr72, Tyr124, Tyr337, Tyr341, Asp74, Trp286, Phe295, and Phe297; Asp74 is responsible for ACh recognition [2]. BChE, different with AChE, hydrolyzes butyrylcholine (BCh), faster than the ACh. AChE inhibitors (AChEIs) increase either level or duration of neurotransmitter activity of ACh at the cholinergic synapse, and other target tissues. AChEI drugs dose-dependently improve several symptoms of AD. Several potent AChEIs such as tacrine, donezepil, galantamine, rivastigmine, and metrifonate, are chemically produced [5]. Inappropriately, they have several drawbacks such as high cost, side effects, low bioavailability, and necessity of blood monitoring [1]. Currently, some AChEIs are isolated from natural resources [6]. Several venoms and related neurotoxins from marine Cnidaria have been revealed as a potential source of bioactive compounds [7,8]. Jellyfish produces various amazing natural products with either toxic or biomedical properties [7]. The jellyfish toxins have been used as a model for the development of new drug promising applications to treat neurodegenerative diseases [8]. Numerous jellyfish venoms are the most promising sources of potent AChE [2, 8], and BChE inhibitors [8].

For the first time, we have previously recorded a population of jellyfish C. andromeda that accrued strangely in bushehr coasts of Iran [9]. Limited experiments have been performed on jellyfish C. andromeda venom and their biological activities [2,7,9-11]. However, their venom components and their mode of actions are still far from our understanding. Nowadays, drug discovery is mostly based on In-silico-chemico-biological approach, and molecular docking plays an influential role in the rational design of drugs [12]. Therefore, it is of interest to study cholinesterases inhibiting compounds from the Cassiopea andromeda venom.

## Methodology

### Materials:

All chemicals and solvents used for extraction and analysis of samples were purchased from Sigma, Merck (Germany), or Fluka chemical companies.

### Preparation of nematocysts:

Specimens of C. andromeda were collected from Nayband bay, in the North (27° 30' S, 52° 35' E) of Bushehr-Iran, and Identity of the species was verified by Professor Brenden Holland from the University of Hawaii [9]. The nematocyst isolation method has been previously described by Nabipour et al. (2018) [2]. Briefly, the tentacles were excised manually from living specimens as soon as possible after capture, and directly placed into small glass containers filled with the third part of seawater and subsequently, carried in the ice bags to our research Centre laboratory. After homogenization (IKA Homogenizer, Germany), kept at 4°C for two days intended for the autolysis of the tissues and release of toxins, then centrifuged (Eppendorf, Germany) at 12,000xg at 4°C for 15 min to exclude the precipitates. The resulting supernatant was lyophilized by freeze-drier (Christ, UK), and kept at -80°C until analysis.

###  Acetylcholinesterase activity in vitro:

**Figure 1:**
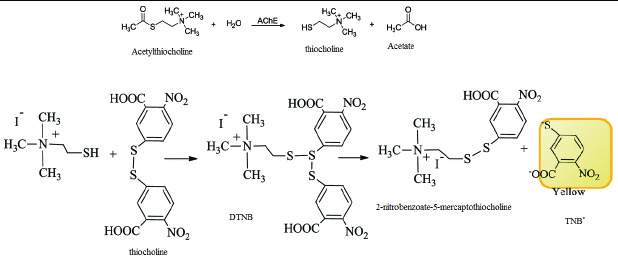
Chemical mechanism of Elman’s method for AChE activity.

The AChE and BChE inhibitory activities of the crude venom were performed according to the Ellman kinetic method, modified by worek et al. (1999) [13]. Acetylthiocholine iodide was applied as a substrate to assay enzyme activities. In this assay, hydrolysis of acetylthiocholine in the presence of the enzyme produces thiocholine. The reduction of the 5, 5'-dithio-bis-2-nitrobenzoic acid (DTNB, Ellman's reagent) to thionitrobenzoate (TNB-) by thiocholine is measured (Figure 1).

### GC-MS analysis of the crude venom:

The lyophilized crude venom was subjected to Young Lin 6900 Gas Chromatography-Mass Spectrometer (YL6900 GC-MS, Gyeonggi-do, South Korea). The identification of compounds was performed by GC-MS, following extraction of the crude venom with methanol: n-Hexane (3:2 v/v). Electron ionization (EI) mass spectra (scan range, m/z 50-500), were taken using electrons of 70 eV energy and with a filament emission of 0.5 mA. GC separations were carried out using an HP-5MS UI column ((30mx0.25mmx0.25µm) i.d., film thickness 0.5µm)). Helium was used as the carrier gas (flow 0.8 ml.min-1). The GC oven was temperature programmed at 5°C min-1 from 80°C after three min since sample injection, and hold at 25°C for 10 min. The injection port of the gas chromatograph, transfer line, and ion source of MSD were maintained at 240, 250, and 270°C, respectively. The separated compounds were identified by matching with compound data in the NIST MS database (2014) library, and the relative (%) amount of each component was measured by comparing its average peak area to the total areas.

### Molecular docking:

**Figure 2:**
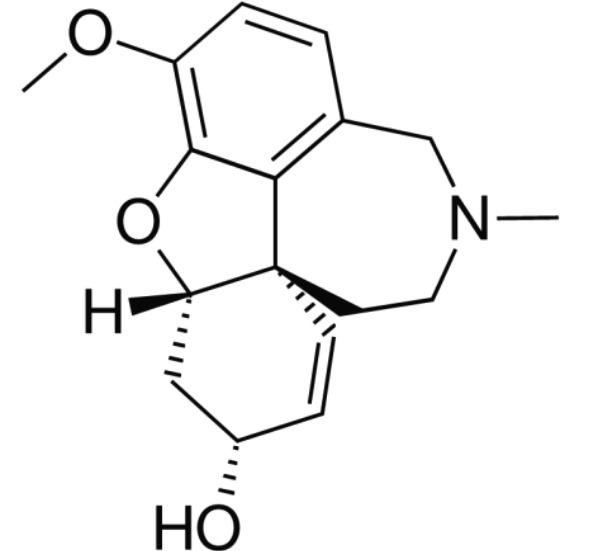
The structure of Galantamine

All identified compounds were screened for their inhibitory effects against AChE and BChE. Galantamine was used as the reference standard (Figure 2). All compounds were energy-minimized with the Gaussian09 program by the DFT method and b3lyp/6-311g basis set[14]. The compound structures were conducted for the docking study against AChE (PDB code: 4BDT) and BChE (PDB code: 4TPK) by Autodock Vina software [15].

### Statistical analysis:

Statistical analysis of data was done using SPSS statistical software version 20. Data were expressed as Mean± SD. Data were compared by one-way ANOVA and p<0.05 was considered significant.

## Results and Discussion:

### Cholinesterase activities:

Experimental data of the crude venom demonstrated promising cholinesterase inhibitory activities against AChE (4.81± 0.25 µM) and BChE (3.5± 0.21µM) at 37°C. In a comparable study, Ayed et al. (2012) indicated the jellyfish Pelagia noctiluca crude venom and its fractions exhibited BChE inhibition activities at different doses without producing acute toxicity [8].

### Gas Chromatography-Mass Spectroscopy of the crude venom:

**Figure 3:**
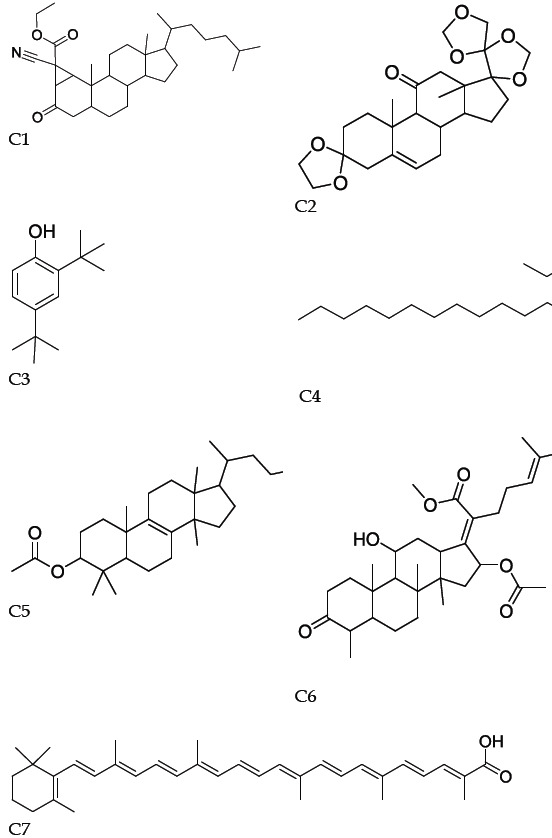
Structure of chemical compositions (C1-C7) obtained from GC-MS analysis of the jellyfish C. andromeda crude venom.

Analysis of the crude venom using the GC-MS has detected seven compounds (C1-C7) with the retention time (RT), 8.01, 9.70, 10.87, 12.64, 13.91, 14.14 and 16.54min, respectively (Figure 3). Patterns were consistent with C1: 3'H-Cycloprop(1,2)-5-cholest-1-en-3-one,1'-carboethoxy-1'-cyano-1,2-dihydro-; (C32H49NO3 (13%)); MW 495., C2: Pregn-5-ene-3,11-dione, 17,20:20,21 bis [methylenebis(oxy)]-, cyclic 3-(1,2-ethane diyl acetal; (C25H34O7 (24%)); MW 446., C3: 2,4-Di-tert-butylphenol; (C14H22O (7%)); MW 206., C4: Octadecane, 3-ethyl-5-(2-ethylbutyl)-; (C26H54 (3%)); MW 366., C5: Acetic acid, 17-(4-hydroxy-5-methoxy-1,5dimethylhexyl)-4,4,10,13,14-penta methyl-2,3,4,5,6,7,10,11,12,13,14,15,16,17- Tetra decahydro cyclo penta[a] phenanthryl ester; (C33H56O4 (37%)); MW 516., C6: 2-(16-Acetoxy-11-hydroxy-4,8,10,14-tetramethyl-3-oxohexadecahydro cyclopenta[a]phenanthren-17-ylidene)-6-methyl-hept-5-enoic acid, methyl ester; (C32H48O6 (37%)); MW 528., C7: 4'-Apo-β,.psi.-carotenoic acid; (C35H46O2 (16%)); MW 498.

### Molecular docking:

Both experimental and rational methods have key roles in the discovery and development of drugs. The docking of biomolecules with the identified structures, even with limited experimental evidence, is developing promptly to become a standard tool for structural biology [12]. Hence, to better understand the experimental results, a molecular docking study was also performed. The affinity values resulted from the docking procedure for AChE and BChE are inferred in the Table 1 (see PDF).

**Figure 4:**
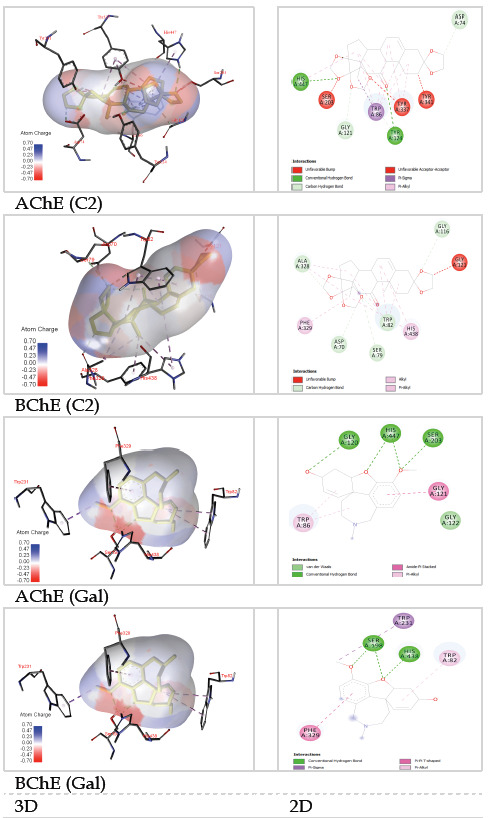
Docking of the compound C2 from Cassiopea andromeda venom and galantamine (as the standard molecule), with AChE, and BChE enzymes (2, and 3-dimensional binding modes).

Binding interaction between a molecule ligand and an enzyme can cause activation or inactivation of the enzyme. Molecular docking displays these interactions, in less time and at negligible cost [12]. According to the Table 1 (see PDF), all compounds (C1-C7) were revealed to be active against AChE and BChE, and their binding energies were greater than that of galantamine. Among the series, compound C2 was found to be the most active against AChE with an affinity value of -9.2 kcal.mol-1. Furthermore, Compounds C1, C6, and C5 were, respectively, found not only to show potent inhibitory effects on AChE but also to be more potent than galanthamine. The AChE inhibition activity of Compound C7 was also observed to be about similar to that of Galanthamine. According to docking results, compound C2 had several strong interactions such as conventional hydrogen, carbon-hydrogen, Alkyl, pi-Alkyl, and van der Waals to numerous amino acid residues that correlated well with the experimental data (Figure 4).

In the case of BChE inhibition studies, all compounds were found to have the ability to inhibit the enzyme. Correspondingly, compound C2 was the most active with an affinity value of -4.6 kcal.mol-1. However, all compounds were found to be less active than the reference compound. The physiological act of BChE is not certainly known. However, it has been found that the amount of enzyme is extensively higher in Alzheimer's plaques than in normal plaques of age-related non-demented brains [16]. Moreover, AChE is known to quicken the aggregation of the β-amyloid peptide during the initial steps of AD. That's why; the administration of inhibitors with different AChE/BChE selectivity may be more helpful in the treatment of AD [17].

**Figure 5:**
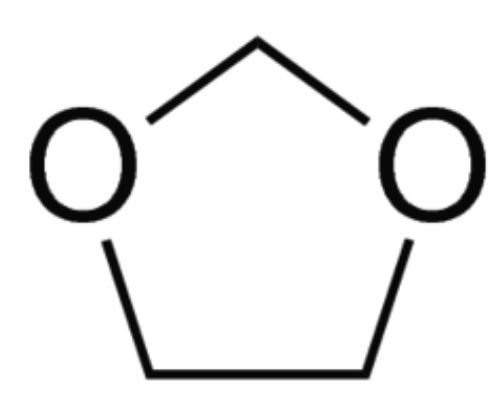
1,3-Dioxolane structure

Theoretical selectivity for AChE is defined as (BChE/AChE), and for BChE is defined as (AChE/BChE) affinities. As perceived, AChE inhibition activities of the venom compounds were approximately twofold greater than for BChE inhibition for the same compounds. Interestingly, compounds C1, C2, C5, and C6 as the most potent inhibitors against AChE and BChE, comprise the steroid skeleton. Compound C1 is a steroidal alkaloid. Numerous studies have provided evidences for the association between steroidal structure and cholinesterase inhibitory activity [17,2]. In a comparable study, Atta-ur-Rahman et al., (2004), have previously isolated five steroidal alkaloids from the ethanolic extract of Sarcococca saligna with cholinesterase inhibitory potential with the IC50 values ranging from 12.5- 200 µM against AChE, and 1.25- 32.2 µM against BChE [18]. The further steroidal compounds such as Haloxysterols A-D, 5,8-epidioxy-(24S)-ethyl-cholest6, 9(11),22(E)-triene-3-ol, (24S)-ethyl-cholest7,9(11),22(E)-triene-3-ol, lawsaritol, and 24ethyl-cholest-7-ene-3,5,6-triol were found to be the most active to moderate AChE inhibitors (IC50 0.89- 26.4 µM) [19]. According to Mohebbi et al. (2018), three compounds, including a neurosteroidal alkaloid androtoxin B, from C. andromeda were potent antiAChE agents with strong binding to both the catalytic and peripheral sites of the enzyme [2]. Among the series of the compounds, compound C2 was selected for molecular docking studies, because of its superior affinity. Compound C2 produced a close contact with two (His447 and Ser203) of the three amino acid residues of the catalytic triad of the enzyme. Also, there were found several van der Waals, carbon-hydrogen, pi-sigma, pi-alkyl, and conventional hydrogen interactions among the compound with the amino acid residues of the enzyme, in the docking study (Figure 4). These steroidal derivatives are the phytochemicals with a widespread range of biological effects including AChEI activity. Moreover, Compound C2 comprises the 1, 3-Dioxolane chemically active ring (Figure 5). Different organic compounds containing the dioxolane ring have shown the antiacetylcholinesterase and cholinomimetic activities [20]. Ungeremine, liriodenine, lycorine, isoquinoline alkaloids stylopine, and lycorine N-oxide with the dioxolane ring were all found to be potent AChE inhibitors. The alkaloids (+)-canadaline and (+)-canadine, both isolated from Corydalis cava, as well as undulatine from Nerine bowdenii, were identified as the significant AChE inhibitors [19]. The presence of the dioxolane ring in compound C2 may be able to contribute to the enzyme inhibitory activity. Overall, the C. andromeda venom constituents more or less demonstrate a good binding affinity towards the active site of the acetyl and butyrylcholinesterases, on the basis of in silico study that correlated well with the experimental data.

## Conclusion

We reported cholinesterases inhibiting compounds from the Cassiopea andromeda venom. The compound C2, a Dioxolane steroid, displayed the strongest inhibition on both AChE and BChE activities for further consideration.
